# Primary Plasma Cell Leukemia Revealed by a Mandibular Lesion: A Case Report

**DOI:** 10.7759/cureus.20148

**Published:** 2021-12-03

**Authors:** Houda Youssefi, Maryame Ahnach, Mounia Bendari, Abderahmane Al Bouzidi

**Affiliations:** 1 Department of Hematology, Cheikh Khalifa International University Hospital, Casablanca, MAR; 2 Faculty of Medicine, Mohammed VI University of Health Sciences, Casablanca, MAR; 3 Department of Pathology, Cheikh Khalifa International University Hospital, Casablanca, MAR

**Keywords:** novel immunomodulatory agents, relapse, extramedullary lesions, plasmacytoma, primary plasma cell leukemia

## Abstract

Primary plasma cell leukemia (PCL) is a rare and aggressive hematological malignancy exhibiting a circulating plasma cell count exceeding 20% of peripheral blood leukocytes or an absolute plasma cell count >2000/mm3. We report a case of a 37-year-old woman presented to the Department of Hematology with a two-month history of growth inside the oral cavity in the upper jaw and weakness. The physical examination revealed a voluminous mass involving the left side of the maxillary gingiva. The maxillofacial computerized tomography (CT) scan confirmed the presence of a solid tissue mass at the left upper maxilla. A biopsy sample obtained from the lesion showed a plasma cell infiltration. The laboratory findings revealed anemia, renal impairment with high levels of creatinine and calcium. Serum protein electrophoresis found a monoclonal peak at IgG lambda, a high level of lambda free light. The diagnosis was subsequently confirmed by a peripheral-blood smear revealed 25% of plasma cells and bone marrow aspiration with 50% of plasma cell infiltration. Primary plasma-cell leukemia (pPCL) was confirmed. The patient received VTD chemotherapy (bortezomib, thalidomide, and dexamethasone) followed by autologous stem cell transplant (ASCT), which resulted in complete remission. At the six-month follow-up, the patient relapsed with extramedullary multiple lesions under ineffective rescue therapy. Response to frontline treatments may be significant initially but short-lived with a dismal median overall survival below one year. This case report aims to highlight the need for awareness among clinicians of the relevance of examining other associated clinical features of pPCL, given its aggressive course and rapid progress without the therapy.

## Introduction

Plasma cell leukemia (PCL) is a rare hematological malignancy classified into primary (pPCL) and secondary (sPCL). pPCL represents the leukemic phase already present at diagnosis in contrast to sPCL, a leukemic progression of a previously diagnosed multiple myeloma (MM). The rising incidence of sPCL is mainly attributed to improved survival in MM, especially with heavily pretreated patients, living long enough for clonal evolution to take place.

According to the original Kyle's criteria, the diagnostic definition of PCL requires both more than 20% circulating plasma cells and an absolute count greater than 2×10^9^/L clonal plasma cells in peripheral blood. However, as stated in the last consensus of the International Myeloma Working Group (IMWG), either one is sufficient for the diagnosis of PCL. The risk of underestimating certain PCLs with a lower degree of peripheral plasmacytosis, such as "early PCL" and "PCL-like" myeloma, suggests a revision of the current diagnostic criteria [1].

Although there has been a survival improvement of pPCL patients as a result of incorporating autologous stem cell transplantation (ASCT) and novel agents into treatment regimens, the prognosis remains poor compared to MM. The reported median overall survival (OS) is as yet below one year [2,3]. In this regard, clinical and genetic characteristics patterns underline PCL and MM as different entities [3].

We hereby present a unique case of a patient diagnosed with pPCL revealed by an oral lesion. Our report highlights the rarity of this uncommon extramedullary involvement in pPCL as an initial manifestation of the disease.

## Case presentation

**Figure 1:**
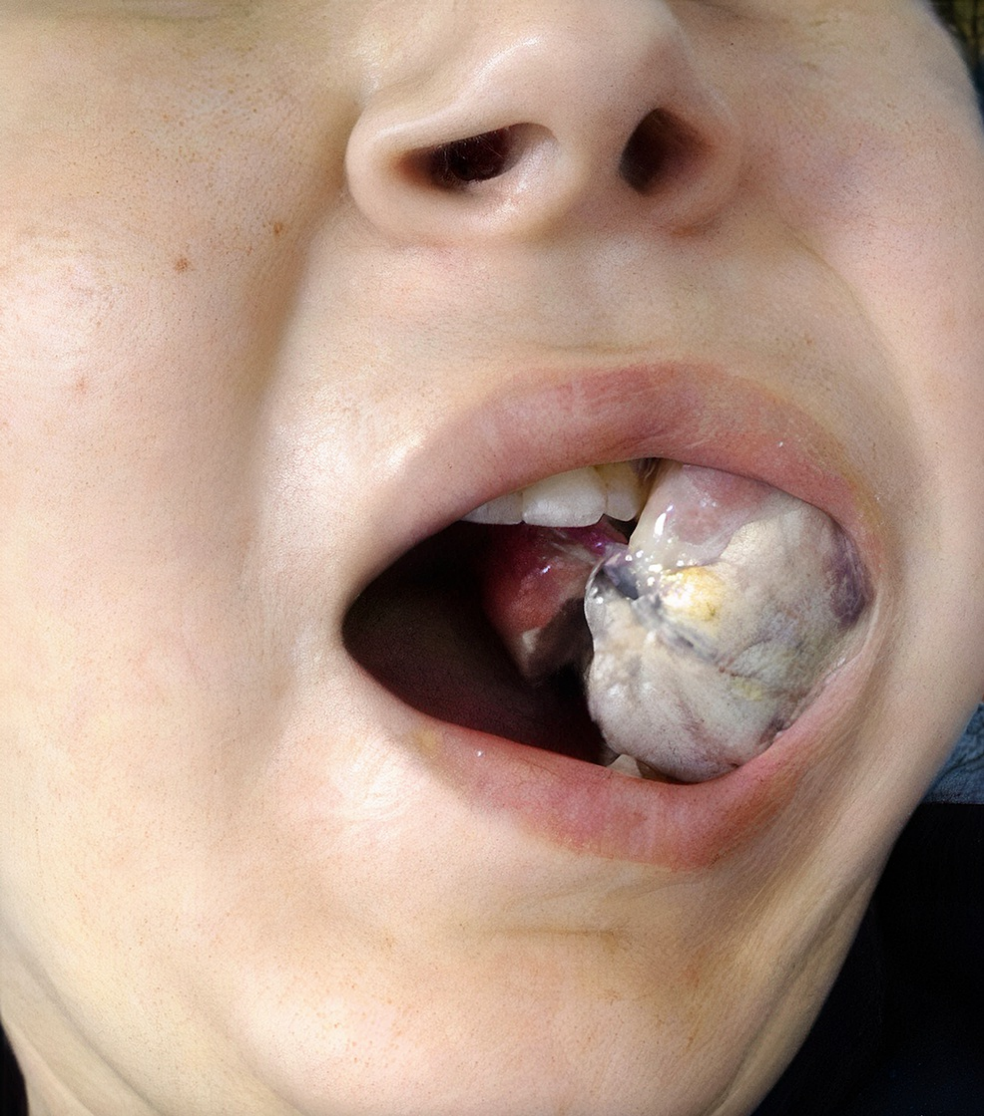
Soft tissue overgrowth involving the left side of the maxillary gingiva, measuring approximately 8 cm

A 37-year-old woman was admitted to the hospital with a two-month history of an overgrowth of soft tissue inside the oral cavity. The patient had no significant personal or family history. For the past 10 days leading up to admission, she reported generalized fatigue, weakness, lethargy, and bone pain. Upon general examination, she was afebrile, normotensive, with pale skin. The intraoral examination revealed a voluminous overgrowth of soft tissue involving the left side of the maxillary gingiva. Most notably, the mass was painless, with a hard consistency measuring approximately 8 cm, non-ulcerated, covered with ill-defined contours (Figure 1) with left laterocervical lymphadenopathy.

The craniofacial and thoracoabdominal computerized tomography (CT) scan confirmed the presence of a left maxillo-jugal mass with a maximum dimension of 10 cm, reaching the alveolar border of the jawbone with tooth loosening and partially filling the lumen of the maxillary sinus. In addition, a pre-renal mass was also present.

**Figure 2:**
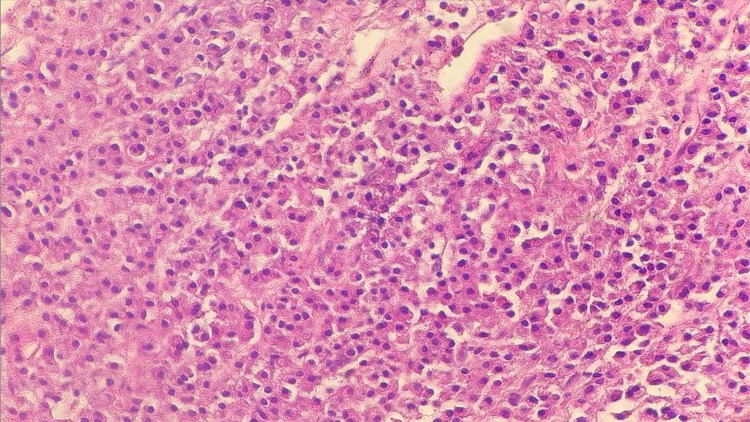
The oral lesion's biopsy shows plasma cell infiltration (HE: G×40) The plasma cells are described as medium-sized cells, plasmacytoid with eccentrically rounded nuclei, patchy chromatin, and abundant eosinophilic cytoplasm.

After obtaining the patient's written informed consent, the mandibular biopsy was performed. The histological examination revealed a massive plasma cell proliferation suggesting a plasmacytoma. Microscopically, the tissue appeared to be massively infiltrated by cellular proliferation with widespread growth, consisting of medium-sized cells, plasmacytoid with eccentrically rounded nuclei, patchy chromatin, and abundant eosinophilic cytoplasm, along with some rare larger cells with hyperchromatic nuclei. The immunohistochemistry analysis on paraffin sections revealed tumor cells with positive immunofixation of CD138+, a weak presence of Kappa and Lambda anti-light chains, and CD20 and CD3 in some reactive lymphocytes, but lacked CD56 positivity (Figure 2).

Initial laboratory investigations portrayed a normocytic normochromic anemia (hemoglobin of about 7.6 g/dL), leukocytosis (total white cell count of 29.73 x 10^3^/microliter), and thrombocytopenia (platelets count of 109 x 10^3^/microliter). The patient also had an acute kidney injury with elevated levels of serum creatinine and urea, respectively of 23.14 mg/L and 0.60 g/L, and lactate dehydrogenase (LDH) 545 IU/L. The twenty-four-hour urine protein was elevated at 10.860 g/24H with a high level of uric acid at 106 mg/l and beta-2 microglobulin at 7.64 mg/L. 

**Figure 3:**
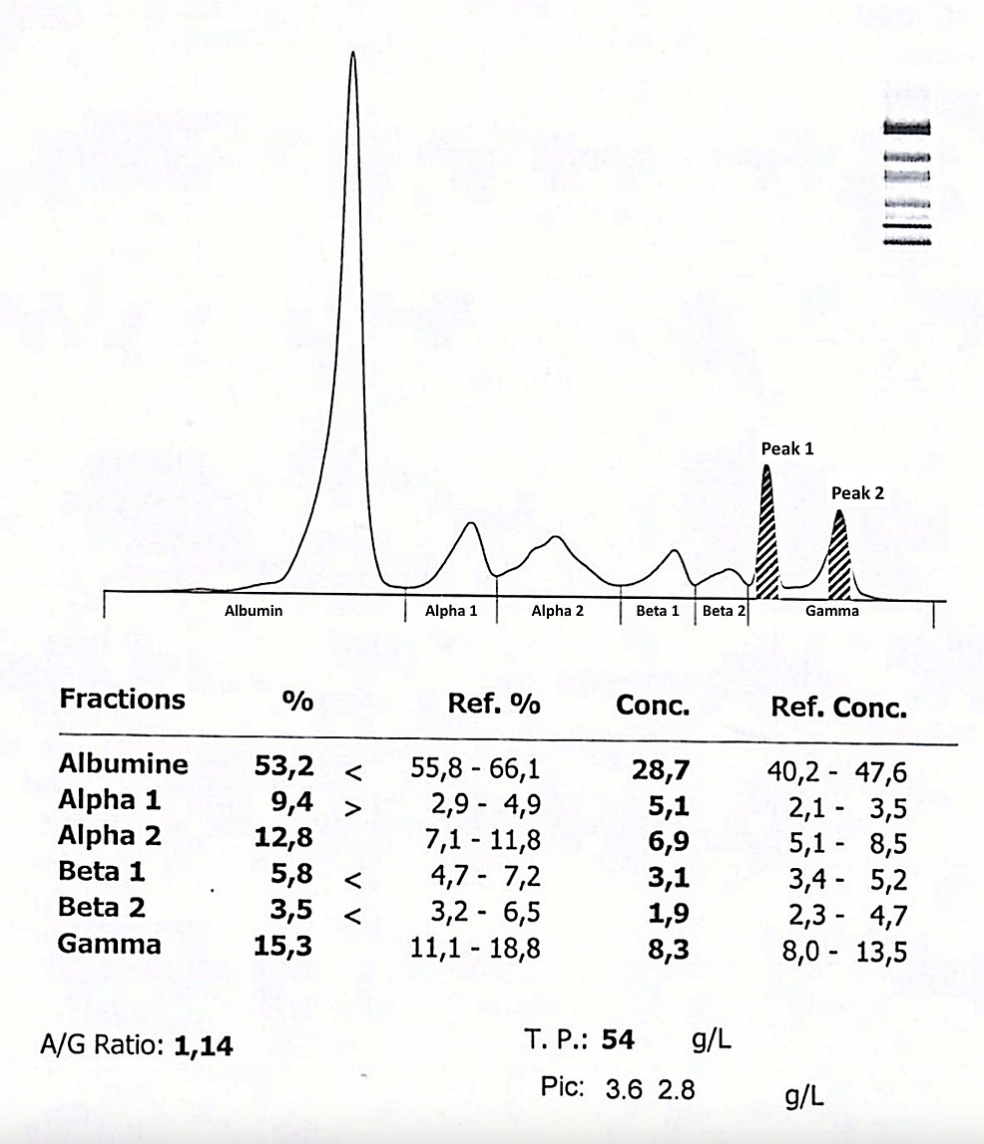
Schematic representation of serum protein electrophoresis The serum protein electrophoresis shows a total protein (T.P) 54 g/L, albumin/globulin (A/G) ratio 1.14, levels of Peaks 1 and 2 respectively of 3.26 g/L and 2.8 g/L and protein fractions in percentage.

**Figure 4:**
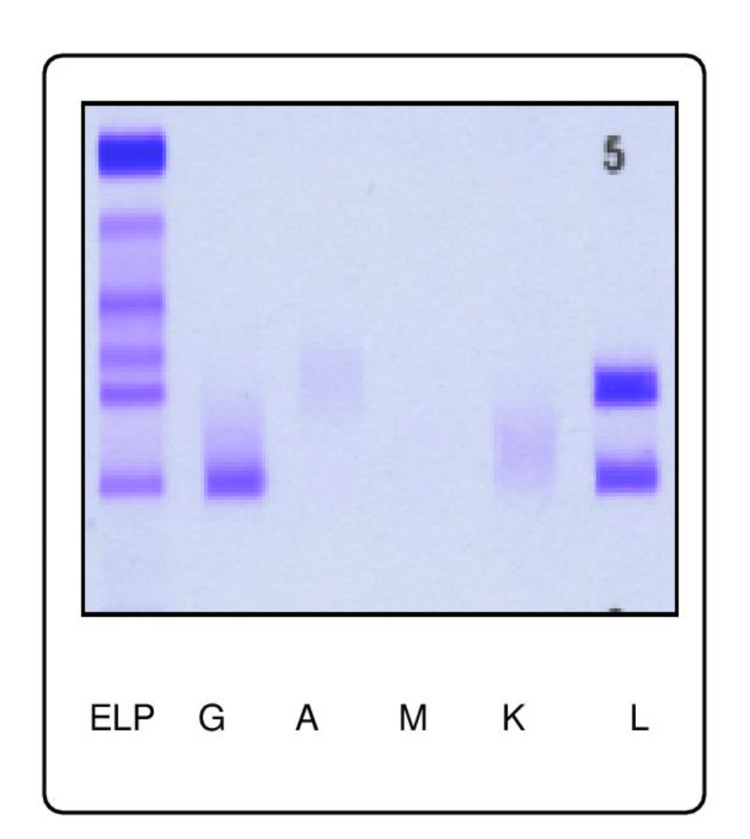
Acid violet staining for immunofixation electrophoresis shows monoclonal IgG lambda paraproteinaemia

Serum protein electrophoresis (Figure 3) and immunofixation (Figure 4) portrayed hypoalbuminemia with biclonal peaks of the gamma-globulin area consisting of a monoclonal band of the IgG Lambda type (Peak 1) associated with an additional monoclonal band revealed with the anti-lambda light chain corresponding to monoclonal lambda free light chains (Peak 2). Also noteworthy, the absence of qualitative abnormality in the delta and epsilon heavy chains. The serum-free light-chain ratio kappa-lambda ratio was equal to 0 with lambda of 14 206 mg/L and kappa of 0.890 mg/L. Urine protein electrophoresis showed the presence of a monoclonal band with an anti-lambda light chain, corresponding to monoclonal lambda free light chains.

**Figure 5:**
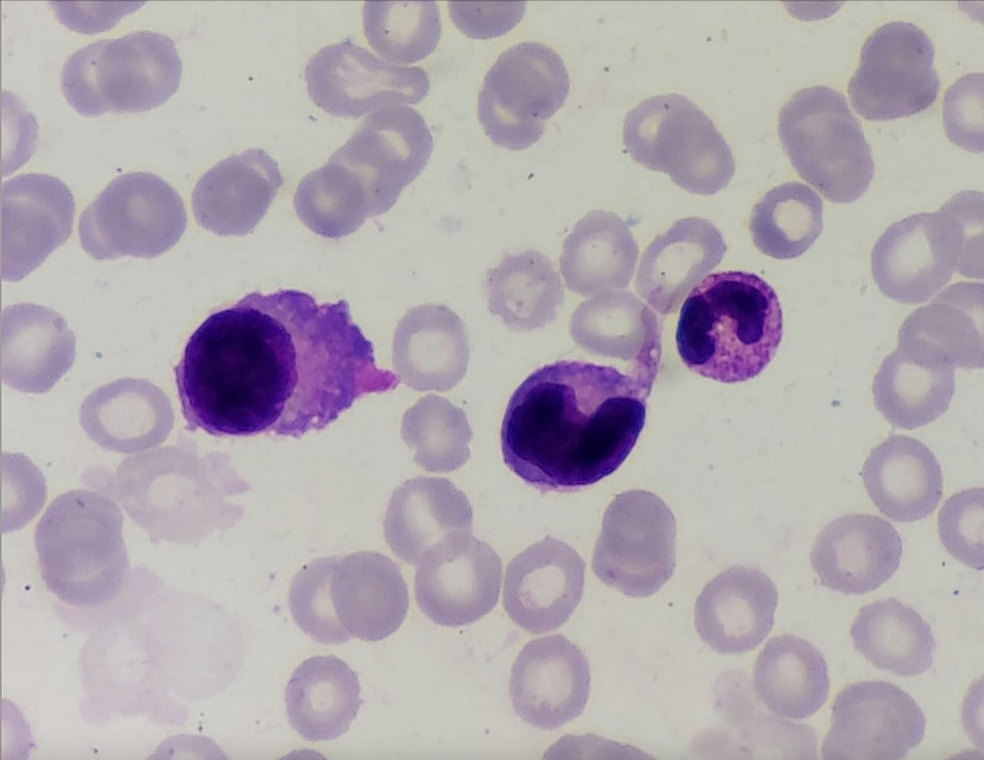
Bone marrow aspirate shows 50% of neoplastic plasma cells

The peripheral blood smear revealed the presence of 25% of plasma cells along with the bone marrow aspiration revealing 50% of plasma cell infiltration (Figure 5).

Additionally, her karyotype was normal. Anti-Hepatitis C, Hepatitis B surface antigen, and Anti-HIV1, two were not detected. 

Given all of the aforementioned and the original Kyle's criteria, a definitive diagnosis of primary plasma cell leukemia was made. The patient received as first-line induction therapy four cycles of the VTD protocol: Velcade® (Bortezomib), 1.3 mg/m^2^ given on days one, four, eight, and 11, daily oral thalidomide 100 mg/day for 21 days continuously and oral dexamethasone 40 mg/day on days one to four and nine to 12. Supportive therapy consisted of hydration, allopurinol, antimicrobial and antithrombotic prophylaxis. After the combination chemotherapy, circulating plasma cells were no longer detectable.

Her repeated peripheral blood film and immunophenotyping did not reveal any plasma cells, nor did the repeated bone marrow morphology immunophenotyping demonstrate any disease. The serum protein electrophoresis and serum-free light chain showed a good partial response to treatment with a normalized renal function. Following this, she underwent an autologous stem cell transplant (ASCT) and was compliant to therapy with no significant adverse effects. After achieving complete remission (CR), the patient left the hospital with forthcoming appointments for the consolidation phase. She was therefore expected to receive, in good clinical conditions, two post-transplantation cycles of the VTD protocol along with thalidomide as maintenance therapy.

Unfortunately, six months after the bone marrow transplant, the patient reported weakness with facial nerve palsy. Her blood work portrayed normochromic normocytic anemia (hemoglobin of about 9.2 g/dL), leukocytosis (total white cell count of 19.49 x 10^3^/microliter), and platelets count of 405 x 10^3^/microliter. Renal impairment was noted with high levels of serum creatinine of 17.60 mg/L and urea 0.47 g/L, along with elevated LDH 333 UI/l, uric acid 85 mg/l, and calcium 191 mg/L. The peripheral blood smear analysis revealed a 50% cluster of neoplastic plasma cells. As for the bone marrow aspiration, 14% dystrophic plasma cell infiltration was noted. Serum protein electrophoresis and immunofixation demonstrated a mild elevation of the first monoclonal peak and the absence of the second peak at the IgG zone.

Thus, the patient was deemed in relapse and received two cycles of the RCD protocol: Revlimid, Endoxan, dexamethasone with a supportive treatment of hypercalcemia consisting of zoledronic acid, dialysis, methylprednisolone, and hydration. After failure of the RCD protocol, a rescue therapy was initiated consisting of two cycles of the TD PACE protocol: dexamethasone with 40 mg, cisplatin 10 mg/m^2^, etoposide 40 mg/m^2^, cyclophosphamide 400 mg/m^2^, doxorubicin 10 mg/m^2^ for four days and daily oral thalidomide for 28 days. The salvage therapy was withheld due to a sudden septic shock leading to her transfer to the intensive care unit. The patient died several days later.

## Discussion

Plasma cell dyscrasias (PCD) include different entities ranging from indolent diseases to aggressive forms characterized by malignant plasma cells leaving the bone marrow. Amidst this spectrum, PCD's most aggressive form: plasma cell leukemia. First established by Noel and Kyle in 1974, the PCL's original definition requires both criteria: the presence of more than 20% circulating plasma cells and an absolute count greater than 2×10^9^/L clonal plasma cells in peripheral blood [4].

There is scant literature regarding PCL's incidence due to its rarity and fulminant nature, and as yet, no formal studies on its occurrence in the general population. However, it accounts for 0.5% to 2% of MM cases, with annual incidence ranging between 0.4 and 1.2 cases per million individuals per year [5]. Unlike MM and sPCL's general population, pPCL is mostly observed in younger patients, with a median age range between 50 and 60 years [1].

It is noteworthy that a correct and timely diagnosis depends on the pathologist's ability to screen and recognize plasma cells in the peripheral blood smear. Many cases of PCL with lower degrees of peripheral plasmacytosis have been reported, suggesting that the current definition underestimates PCL's frequency and thereby delays early intervention and management strategies. In addition, the original Kyle's criteria do not include investigating the clonality of plasma cells as their presence in the peripheral blood is not only limited to pPCL. Given all the above, revisiting the definition and identifying the correct threshold for circulating plasma cells is needed [6-9]. Both the IMWG and WHO suggest that only one of the two criteria is sufficient for the diagnosis [3]. Aside from pPCL, low circulating plasma cells comprising 5% or more of the peripheral blood white cell differential count can also be found transiently in other non-malignant conditions such as mononucleosis, severe infections including or serum sickness [6].

Herein, our patient's diagnosis was initially revealed by a mandibular lesion. It is of note that oral lesions are substantially rare sites in pPCL, especially as the initial clinical manifestation of the disease [4-6]. The diagnosis of pPCL was confirmed according to Kyle's criteria and given all the above-stated results of the mandibular biopsy, the peripheral blood smear, and bone marrow aspiration.

The extramedullary involvement in pPCL is frequently found in the liver, spleen, body cavity effusion, and spinal fluid, in contrast to osteolytic lesions [10]. The latter is presumably due to plasma cells leaving the bone marrow towards the peripheral circulation before forming bone tumors [11]. This leukemic expansion is favored by a lower expression of cell adhesion molecules, such as CD56, facilitating the release of leukemic plasma cells from the bone marrow microenvironment [12]. Moreover, the anchoring molecule CD56 is more frequently found to be positive in MM, unlike the B cell marker CD20, more often found in PCL. Despite the mildly different immunophenotype of PCL and MM, the expression of either CD138 or CD38 does not differ between the two groups [3].

Several molecular and genetic profilings demonstrated an elevated genomic instability in pPCL with several cytogenetic aberrations and other molecular lesions at diagnosis, yet, our patient's karyotype was normal. The most chiefly reported alterations are t(11;14) as well as some abnormalities in chromosomes one and 17 [1].

Additionally, serum and urine protein electrophoresis with immunofixation remain essential to determine the clonal nature of plasma cells. Likewise, our patient's results portrayed a biclonal peak of the gamma-globulin area consisting of a monoclonal band of the IgG Lambda type and a monoclonal lambda free light chains. This finding is most prominently a rare occurrence in patients with pPCL [10].

The clinical presentation of pPCL typically includes the CRAB symptoms : hypercalcemia (Ca ≥12 mg/dL), renal insufficiency (Cr ≥2.0 mg/dL), anemia (Hb <10 g/dL) and bone lesions. Equally, our patient presented three of these clinical features: normocytic normochromic anemia with hemoglobin levels of about 7.6 g/dL, acute kidney injury with an elevated creatinine at 23.14 mg/L, and hypercalcemia at 191 mg/L. Overall, patients with pPCL are frequently afflicted with renal insufficiency and elevated β2-microglobulin compared to diagnosed MM, which aligns with our patient's clinical findings. However, they do not have clinical evidence of overt bone destruction as opposed to MM and sPCL patients [8-10].

This patient's course also underlines a twofold conundrum: on a prime level, a prevailing inefficiency of salvage therapies in refractory pPCL along with an abiding poor prognosis, notwithstanding advances in novel treatments and ASCT. To date, pPCL's prognosis remains dismal, with a median survival of seven months [9]. In 2018, a multicenter retrospective study of 117 patients suggested a prognostic score based on age ≥60 years, platelet count ≤100 × 10^9^/l, and peripheral blood plasma cell count ≥20 × 10^9^/l as predictors of worse survival. However, this score is still in the progress of validation [1,3,13,14,15].

The introduction of novel agents such as the proteasome inhibitor bortezomib and the immunomodulatory drugs (IMID) has dramatically improved the outcome of patients with MM in contrast to pPCL patients with rather conflicting results and no significant outcome [2]. In addition to this, a large study conducted by the European Group for Blood and Marrow Transplantation has reported that PCL patients were more likely to enter complete remission. Yet, the response was short-lived with a markedly increased non-relapse-related mortality compared to MM patients [16].

To date, available data still recommends the prompt use of bortezomib-containing regimens followed by high-dose chemotherapy and ASCT as a first-line treatment. This approach is only endorsed in young patients under 75 years with a good clinical condition. Conversely, the European myeloma network recommendations on diagnosis and management of patients with pPCL reported that the use of aggressive chemotherapy protocols including hyper-CVAD-VD and VTD/VRD-PACE remains with no clear evidence of their superiority [14]. Similarly, our patient was eligible for bortezomib-based protocols such as the VTD-PACE given her young age. However, her clinical frailty and lethargic state, along with acute renal impairment, had precluded the use of the latter. The patient, therefore, received four cycles of the VTD protocol followed by ASCT and verily achieved complete remission.

As for salvage therapies, the protocol choice depends on the type and response to previous ones. Clinically eligible patients may be candidates for bortezomib-based regimens or intensive chemotherapy, but results after treatment remain unsatisfactory [14]. Comparatively, our patient received two rescue therapy protocols: RCD followed by TD-PACE, both deemed unsuccessful. Likewise, results after chemotherapy of ineligible patients for transplant procedures appear to be disappointing due to advanced age and frailty.

Several new treatment modalities are currently being tested, focusing merely on reducing early mortality and achieving long-term disease control with efficacious agents. Amongst these emerging therapies is venetoclax, a BCL-2 inhibitor, used in combination with daratumumab, dexamethasone, and bortezomib in refractory PCL patients harboring the t(11;14). Used as a first treatment cycle, this particular molecule has so far shown early and deep response [17]. Amidst the new IMIDs is pomalidomide, a third-generation molecule resulting in improved overall survival in refractory MM and PCL [18].

Encouraging reports also arise from the use of ixazomib and carfilzomib-based regimens. These second-generation proteasome inhibitors are presently being tested as a maintenance treatment after ASCT in relapsed MM and PCL patients and as an induction treatment in PCL, respectively. Antibodies such as daratumumab, an anti-CD38, have also shown impressive results in terms of efficacy in refractory MM. Lastly, the CAR-T therapy, a new technique using genetically engineered autologous T-cells, is also currently being developed for MM and PCL patients [19].

## Conclusions

Plasma cell leukemia is a rare distinct clinical-pathologic entity with a dismal prognosis. A thorough medical history, radiological and histopathological examination weighted all equally are crucial for an early diagnosis. Our case report highlights the rarity of oral lesions as a first clinical feature of this condition. Uncommon extramedullary manifestations such as mandibular lesions may hinder early diagnosis, as demonstrated in our patient's case. Clinicians must therefore ponder upon it for early detection and treatment. 
